# Virulence-determinants and antibiotic-resistance genes of MDR-*E. coli* isolated from secondary infections following FMD-outbreak in cattle

**DOI:** 10.1038/s41598-020-75914-9

**Published:** 2020-11-13

**Authors:** Abdelazeem M. Algammal, Helal F. Hetta, Gaber E. Batiha, Wael N. Hozzein, Waleed M. El Kazzaz, Hany R. Hashem, Ayat M. Tawfik, Reham M. El-Tarabili

**Affiliations:** 1 Department of Bacteriology, Immunology, and Mycology, Faculty of Veterinary Medicine, Suez Canal University, Ismailia, 41522 Egypt; 2 Department of Medical Microbiology and Immunology, Faculty of Medicine, Assuit University, Assuit, 71515 Egypt; 3 Department of Internal Medicine, College of Medicine, University of Cincinnati, Cincinnati, OH 45267-0595 USA; 4 Department of Pharmacology and Therapeutics, Faculty of Veterinary Medicine, Damanhour University, Damanhour, 22511 AlBeheira Egypt; 5 Bioproducts Research Chair, Zoology Department, College of Science, King Saud University, Riyadh, 11451 Saudi Arabia; 6 Botany and Microbiology Department, Faculty of Science, Beni-Suef University, Beni-Suef, 62511 Egypt; 7 Botany Department, Faculty of Science, Suez Canal University, Ismailia, 41522 Egypt; 8 Department of Microbiology and Immunology, Faculty of Pharmacy, Badr University in Cairo, Cairo, 11829 Egypt; 9 Public Health and Community Medicine Department, Faculty of Medicine, Port-Said University, Port-Said, 42526 Egypt

**Keywords:** Bacteriology, Microbiology, Infectious-disease diagnostics

## Abstract

This study aimed to evaluate the prevalence, multidrug-resistance traits, PCR-detection of virulence, and antibiotic-resistance genes of *E. coli* isolated from secondary infections following FMD-outbreak in cattle. A total of 160 random samples were gathered from private dairy farms in Damietta Province, Egypt. The specimens were subjected to bacteriological examination, serotyping, congo-red binding assay, antibiogram-testing, and PCR-monitoring of virulence-determinant genes (*tsh*, *pho*A, *hly*, *eae*A, *sta*, and *lt*) as well as the antibiotic-resistance genes (*bla*_TEM_, *bla*_KPC_, and *bla*_CTX_). The prevalence of *E. coli* was 30% (n = 48) distributed in 8 serogroups (40/48, 83.3%), while 8 isolates (8/48, 16.6%) were untypable. Besides, 83.3% of the examined isolates were positive for CR-binding. The tested strains harbored the virulence genes *pho*A, *hly*, *tsh, eae*A, *sta*, and *lt* with a prevalence of 100% and 50%, 45.8%, 25%, 8.4%, and 6.2%, respectively. Furthermore, 50% of the recovered strains were multidrug-resistant (MDR) to penicillins, cephalosporins, and carbapenems, and are harboring the *bla*_TEM_, *bla*_CTX_, and *bla*_KPC_ genes*.* Moreover, 25% of the examined strains are resistant to penicillins, and cephalosporins, and are harboring the *bla*_TEM_ and *bla*_CTX_ genes. To the best of our knowledge, this is the first report concerning the *E. coli* secondary bacterial infections following the FMD-outbreak. The emergence of MDR strains is considered a public health threat and indicates complicated treatment and bad prognosis of infections caused by such strains. Colistin sulfate and levofloxacin have a promising in vitro activity against MDR-*E. coli*.

## Introduction

Foot and mouth disease (FMD) is a primary contagious disease of a significant threat to ruminants^1, 2^. Globally, it causes severe financial loss in the veterinary sector owing to the high cost of treatment, vaccination, and production losses^3, 4^. Recently, three common strains, A, O and SAT 2 are endemic in Egypt. Despite the routine application of vaccination programs in Egypt, a high prevalence of FMD-outbreaks was recorded^5^. The FMD-Vaccination is usually accompanied by immunosuppression that may lead to secondary bacterial infections in the vaccinated animals^3^. *Escherichia coli* (*E. coli*) is an opportunistic microorganism that is usually inhabitants of the intestinal tract of both humans and animals. *E. coli* represents a common bacterial pathogen which incriminated in various secondary infections^6^. The pathogenicity of *E. coli* is governed by several virulence factors such as; hemolysins, enterotoxins, Shiga-toxins, intimin, fimbria-mannose binding type1-H adhesion, alkaline phosphatase, and Temperature Sensitive Haemagglutinin (Tsh-protein) which are encoded by the specific virulence genes: *hly, lt, sta, stx1, stx2, eae*A*, fim*H*, pho*A*,* and *tsh,* respectively^7, 8^.

Concerning the site of infection, *E. coli* is categorized into (1)-intestinal pathogenic *E. coli*, and (2)-extra-intestinal pathotype. Moreover, Virulent *E. coli* strains, which usually affect both animals and humans, are categorized in various pathotypes according to the mechanism of disease occurrence, including; Enterotoxigenic, Enteropathogenic, Enteroinvasive, Enteroaggregative, and Shiga-toxigenic pathotypes^9^. Enterotoxigenic *E. coli* is the main pathotype that incriminated in white scour in calves, both enterotoxins (heat-labile and heat-stable) and fimbrial-adhesions govern the pathogenesis of the disease. Although Enteropathogenic *E. coli* doesn't produce enterotoxins, it causes severe watery diarrhea in cattle by other mechanisms. Briefly, the bacteria do intimate-adhesion (non-fimbrial adhesion called intimin) with the enterocyte apical cell membrane resulting in the demolition of the intestinal brush border. Furthermore, the Enteroinvasive *E. coli* can invade the epithelial cells of the large intestine, causing ulcerations and inflammation. The invasion process is controlled by a specific plasmid (140 MDa) encoding for the release of various outer membrane proteins involved in the disease pathogenesis^10, 11^.

Globally, the β-lactam antibiotics (cephalosporins, carbapenems, and penicillins) represent about 60% of the used antimicrobial agents^12–14^. The emerging multidrug-resistant *E. coli* is considered a public health threat. The antimicrobial resistance in *E. coli* is mainly attributed to the Extended-Spectrum Beta-Lactamases (ESBLs); which could destroy various β-lactam antimicrobial agents as penicillins, various generations of cephalosporins, and carbapenems^15^. ESBLs are encoded by specific ESBL-genes such as; *bla*_TEM_ (encoded for penicillins-resistance), *bla*_KPC_ (encoded for carbapenems-resistance), and *bla*_CTX_ (encoded for cephalosporins-resistance). The emergence of multidrug-resistant virulent *E. coli* has been described by previous studies^16–18^.

This study was performed to inspect the prevalence, antibiogram, PCR detection of virulence genes (*tsh*, *pho*A, *hly*, *eae*A, *sta*, and *lt*) as well as the antibiotic-resistance genes (*bla*_TEM_, *bla*_KPC_, and *bla*_CTX_) of *E. coli* isolated from secondary bacterial infections following FMD-outbreak in cattle.

## Methods

### Animal ethics

All methods performed consistent with relevant guidelines and regulations. Well-trained experts conducted the handling of animals and experimental procedures. Handling of animals and all protocols were approved by the Animal Ethics Review Committee of Suez Canal University (AERC-SCU), Egypt.

### Sampling and clinical examination

One hundred and sixty specimens; milk (n = 40), blood (n = 40), fecal swabs (n = 40), and nasal swabs (n = 40) were randomly collected under complete aseptic conditions from two private cattle farms (native breeds cows of both sexes with average two years old age and with a history of FMD-outbreak) at Damietta Province, Egypt (From March 2019 to August 2019). The sampling was carried out after FMD-outbreak. The examined farms are very close to each other and sharing the same management practices, nutrition, and water supply. The sampling was performed according to the clinical signs. Blood specimens were gathered from animals suffering from fever, milk specimens were collected from clinically mastitic animals, fecal swabs were collected from diarrheic animals, and nasal swabs were collected from animals that exhibited respiratory manifestations. The examined animals were previously treated with trimethoprim and amoxicillin without improvement. The obtained specimens were processed as soon as possible at the same day of collection and were collected on tryptic soy broth (Oxoid, Hampshire, UK).

### Isolation and identification of *E. coli* and other pathogens

For isolation of *E. coli*, swabs from the obtained specimens were inoculated in McConkey's broth (Oxoid, Hampshire, UK), followed by incubation for 24 h at 37 °C. A loopful of broth-culture was streaked onto MacConkey's agar, and eosin methylene blue agar (Oxoid, Hampshire, UK). The suspected colonies were identified according to their colonial characters, hemolytic activity, microscopical examination using Gram's staining, motility test, hemolytic activity on blood agar, and biochemical reactions (oxidase, catalase, indole, lactose fermentation, methyl-red, citrate-utilization, H_2_S, Voges-Proskauer, and urease tests) as described by Quinn^19^.

For isolation of other bacterial pathogens, swabs from the processed specimens were inoculated on nutrient agar, blood agar, mannitol salt agar, cetrimide agar, and MacConkey's agar (Oxoid, Hampshire, UK), then the inoculated plates were incubated for 24–48 h at 37 °C. The obtained pure colonies were identified according to their colonial characters, morphological characters, and biochemically as described by Quinn^19^.

### *E. coli* serotyping

The retrieved isolates were serotyped for somatic antigen (O-antigen) by the aid of slide agglutination test using standard polyvalent and monovalent commercial *E. coli* antisera (Denka Seiken-Co., Ltd., Tokyo, Japan) at the Animal-Health Research-Institute, Dokki, Egypt as described by Starr^20^.

### Congo-red binding

To emphasize the pathogenicity and the invasiveness of the isolated strains, the assessment of congo-red binding was performed on trypticase agar (containing 0.03% CR dye) (Oxoid, UK). The tested strains were inoculated on trypticase agar and then incubated at 37 °C for 24 h. Then plates were preserved at room temperature (for 48 h). The positive result is indicated by the appearance of red colonies as previously reported by Panigrahy and Yushen^21^.

### Antimicrobial susceptibility testing

The recovered *E. coli* strains were assessed for their antimicrobial resistance using the disc diffusion method on Mueller–Hinton agar (Oxoid, UK). The following antimicrobial agents were involved; ampicillin (AMP) (10 μg), meropenem (MEM) (10 μg), amikacin (AK) (30 μg), trimethoprim–sulfamethoxazole (SXT) (19:1 μg), imipenem (IMP) (10 μg), amoxicillin–clavulanic acid (AMC) (30 μg), ceftazidime (CAZ) (30 μg), cefotaxime (30 μg) (CTX), levofloxacin (LEV) (5 μg), amoxicillin (AMX) (10 μg), and colistin sulfate (CT) (10 μg) (Oxoid, Basingstoke, UK). The *E. coli*-ATCC 25922 was used as a reference strain. Zone-diameters were interpreted according to CLSI^22^. The tested antibiotics are the most commonly used antimicrobial agents in Egypt in both veterinary and health sectors. The tested strains are classified into MDR, XDR and PDR as previously described by Magiorakos^15^.

### Molecular typing of virulence-determinant genes and antibiotic-resistance genes

PCR-monitoring of virulence-determinant genes (*tsh*, *pho*A, *hly*, *eae*A, *sta*, and *lt*) and the antibiotic-resistance genes (*bla*_TEM_, *bla*_KPC_, and *bla*_CTX_) was carried out. The selection of these antibiotic-resistance genes was based upon the results of the antimicrobial susceptibility testing, moreover, the selection of the current virulence genes is based upon their significant role in the pathogenesis of the disease as described in previous studies^9, 10, 18^. Genomic DNA of the examined strains was extracted regarding the manufacturer's guidelines of the QIAamp DNA Mini Kit (Qiagen, GmbH, Germany/Catalogue No.51304). The reaction volume was adjusted at 25-μl (3 μl of genomic-DNA, 5 μl of 5 × Master Mix, and 20 pmol of each prime, the reaction volume was completed by adding distilled H_2_O). Positive controls (provided by A.H.R.I, Egypt) and negative controls (DNA-free) were used in all reactions. The sequences of the used primers (Metabion International AG, Germany) and the PCR-cycling conditions are illustrated in Table 1. Finally, the separation of the obtained products was performed using the agar gel electrophoresis (1.5% agarose stained with ethidium bromide 0.5 μg/ml), and the gel was photographed.

**Table 1:** Oligonucleotides sequences, target genes, specific amplicon size, and PCR re-cycling conditions.

Target gene	Primers sequences	Amplicon size (bp)	Amplification (35 cycles)			References
			Denaturation	Annealing	Extension	
*lt*	GGTTTCTGCGTTAGGTGGAA	605	94 °C 30 s	57 °C 45 s	72 °C 45 s	^23^
	GGGACTTCGACCTGAAATGT					
*sta*	GAAACAACATGACGGGAGGT	299	94 °C 30 s	57 °C 30 s	72 °C 30 s	^23^
	GCACAGGCAGGATTACAACA					
*eae*A	ATGCTTAGTGCTGGTTTAGG	248	94 °C 30 s	51 °C 30 s	72 °C 30 s	^24^
	GCCTTCATCATTTCGCTTTC					
*tsh*	GGT GGT GCA CTG GAG TGG	620	94 °C 30 s	54 °C 40 s	72 °C 45 s	^25^
	AGT CCA GCG TGA TAG TGG					
*pho*A	CGATTCTGGAAATGGCAAAAC	720	94 °C 30 s	60 °C 40 s	72 °C 1 min	^26^
	CGTGATCAGCCCTGACTATGAC					
*hly*	AACAAGGATAAGCACTGTTCTGGCT	1177	94 °C 30 s	54 °C 40 s	72 °C 45 s	^27^
	ACCATATAAGCGGTCATTCCCGTCA					
*bla*_KPC_	ATGTCACTGTATCGCCGTCT	882	94 °C 1 min	55 °C 1 min	72 °C 1 min	^28^
	TTACTGCCCGTTGACGCCC					
*bla*_CTX_	ATG TGC AGY ACC AGT AAR GTK ATG GC	593	94 °C 30 s	54 °C 40 s	72 °C 45 s	^29^
	TGG GTR AAR TAR GTS ACC AGA AYC AGC GG					
*bla*_TEM_	ATCAGCAATAAACCAGC	516	94 °C 30 s	54 °C 40 s	72 °C 45 s	^30^
	CCCCGAAGAACGTTTTC					

### Statistical analyses

The Chi-square test was performed to analyse the obtained results (SAS software, version 9.4, SAS Institute, Cary, NC, USA) (significance level; *P* < 0.05). Furthermore, the correlation analysis was conducted using R software (version 4.0.2; https://www.r-project.org/), it was calculated using the “cor” function and visualization using the “corrplot” functions from the “corrplot” package.

## Results

### Prevalence of *E. coli* and other bacterial pathogens in the examined animals

**Figure 1:**
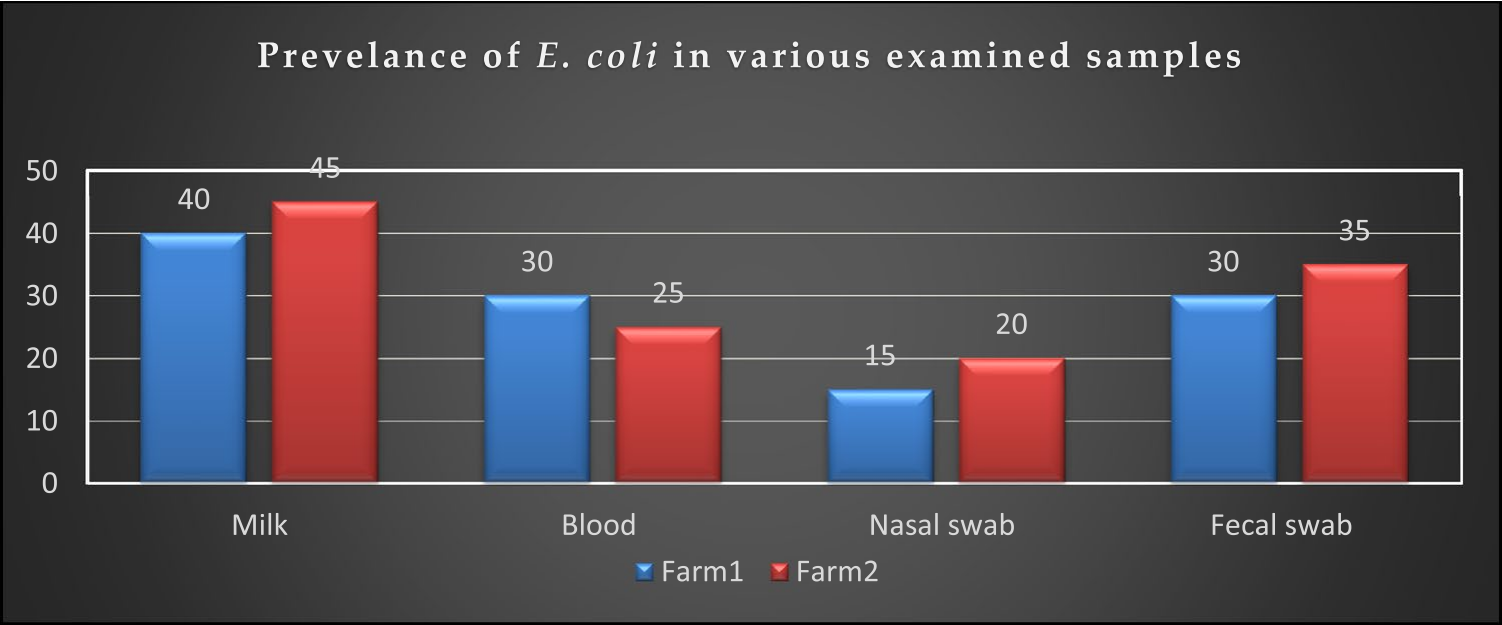
Prevalence of *E. coli* in various examined samples. The prevalence of *E. coli* was 42.5%, 27.5%, 17.5%, and 32.5% in the examined milk samples, blood specimens, nasal, and fecal swabs, respectively.

Regarding the phenotypic characteristics of the retrieved *E. coli*; isolates were identified as *E. coli* based on their morphology and biochemical characteristics. Microscopically, the bacteria appeared as Gram-negative moderate size, motile, and non-sporulated rods. The bacteria grew well on MacConkeys's agar and gave characteristic pink colonies due to lactose fermentation. On blood agar, the colonies are hemolytic, moreover on EMB; the bacteria gave characteristic metallic sheen colonies. Biochemically, all isolates were positive for catalase, lactose fermentation, indole, and methyl-red, tests. Simultaneously, they were negative for cytochrome oxidase, Voges-Proskauer, citrate-utilization, H_2_S production, and urease tests. The bacteriological inspection proved that the total prevalence of *E. coli* was 30% (48/160); the prevalence of *E. coli* was 28.75% (23/80) in the farm (1), while it was 31.25% (25/80) in the farm (2) as described in Table 2. Concerning the types of the tested samples, the prevalence of *E. coli* was 42.5%, 27.5%, 17.5%, and 32.5% in the examined milk samples, blood specimens, nasal, and fecal swabs, respectively (Table 2, Fig. 1). Statistically, there is no significant difference in the prevalence of *E. coli* between the examined farms (*P* > 0.05).

**Table 2:** Prevalence of *E. coli* in various types of samples obtained from diseased cattle.

Examined samples	Number and percentage of *E. coli*					
	Farm 1 *n* = 80		Farm 2 *n* = 80		Total	
	N	%	N	%	N	%
Milk	8/20	40	9/20	45	17/40	42.5
Blood	6/20	30	5/20	25	11/40	27.5
Nasal swabs	3/20	15	4 /20	20	7/40	17.5
Fecal swabs	6/20	30	7/20	35	13/40	32.5
Total	23/80	28.75	25/80	31.25	48/160	30

Chi-square value = 3.112, *P* value = 0.375.

Besides, 70% of the examined diseased animals (n = 112) are infected with other bacterial pathogens including; in mastitis: *Streptococcus uberis* (10/40, 25%), *Streptococcus bovis* (8/40, 20%) and* Enterococcus faecalis* (5/40, 12.5%), in fever: *Pseudomonas aeruginosa* (10/40, 25%), and *Mannheimia hemolytica* (6/40, 15%), in respiratory manifestations: *Pasturella multocida* (10/40, 25%), *Mannheimia hemolytica* (4/40, 10%), and *Pseudomonas aeruginosa* (4/40, 10%), and in diarrhea; *Proteus mirabilis* (17/40, 42.5% ) and *Enterococcus faecalis* (10/40, 25%).

### Serotyping of the recovered *E. coli* isolates

**Figure 2:**
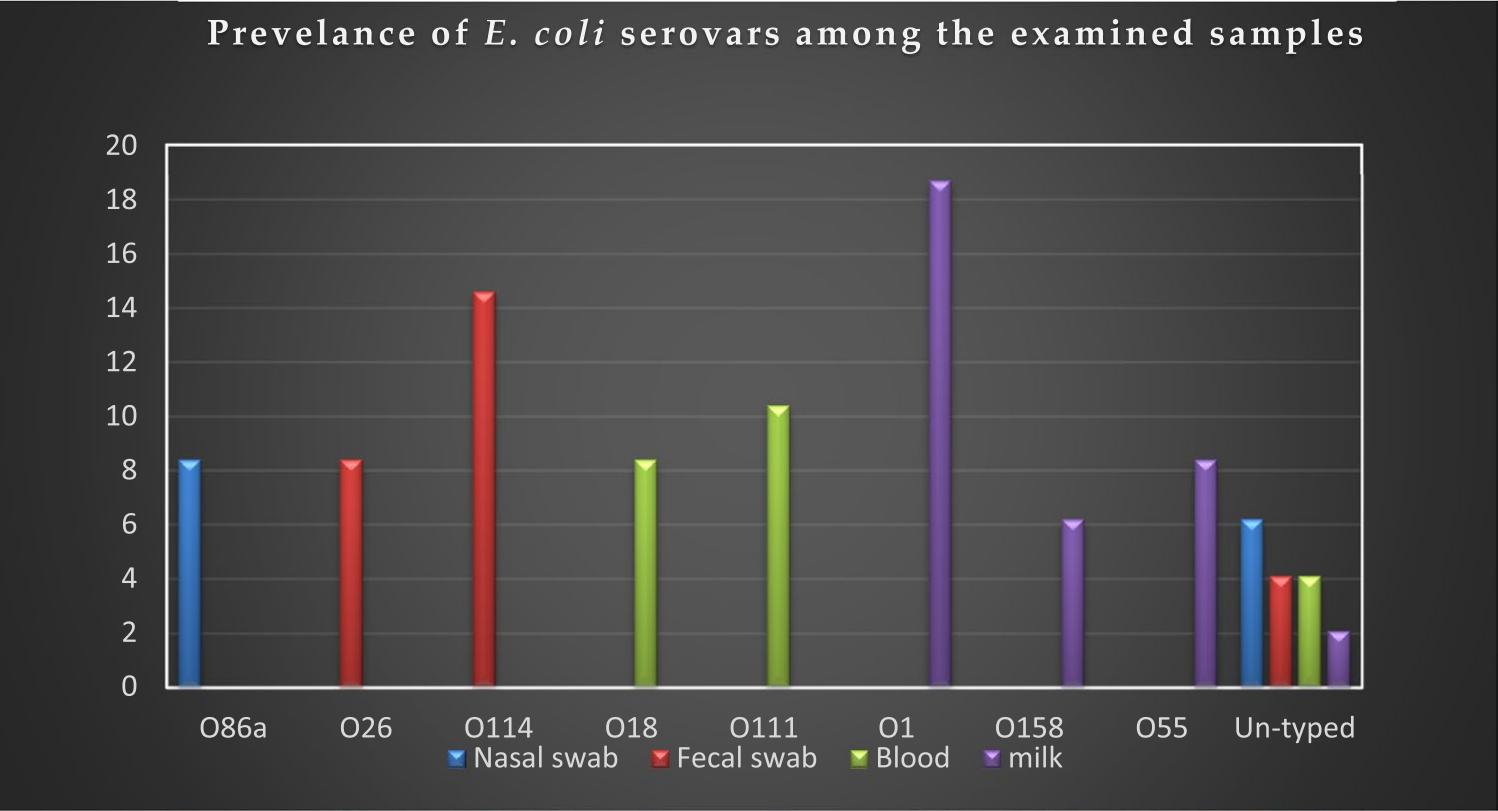
The distribution of *E. coli* serovars among various examined samples. The most prevalent *E. coli* serovar accompanied the respiratory infection was 086a, diarrhea: O114, fever: O111, and mastitis: O1.

The serotyping of the retrieved isolates showed that 40 isolates belonged to 8 O-serogroups and were distributed as the following: O1 (9/48, 18.7%), O114 (7/48, 14.6%), O111 (5/48, 10.4%), O18 (4/48, 8.4%), O26 (4/48, 8.4%), O55 (4/48, 8.4%), O86a (4/48, 8.4%), and O158 (3/48, 6.2%). Furthermore, the remaining isolates (8/48, 16.6%) were untypable (Table 3; Fig. 2). Regarding the type of the examined samples, the *E. coli* serovars scattered as the following; nasal swabs: O86a (4/48) and untyped strains (3/48), fecal swabs: O114 (7/48), O26 (4/48), and untyped strains (2/48), blood samples: O111 (5/48), O18 (4/48), and untyped strains (2/48), milk samples: O1 (9/48), O55 (4/48), O158 (3/48), and untyped strains (1/48). Statistically, there is a significant difference in the prevalence of different serovars retrieved from various types of samples (*P* < 0.05).

**Table 3:** Prevalence of *E. coli* serovars isolated from the examined diseased cattle.

Sample-types	Serovars	*Number*	%
Nasal swabs	086a	4	8.4
	Untyped	3	6.2
Fecal swabs	O114	7	14.6
	O26	4	8.4
	Untyped	2	4.1
Blood-samples	O111	5	10.4
	O18	4	8.4
	Untyped	2	4.1
Milk samples	O1	9	18.7
	O55	4	8.4
	O158	3	6.2
	Untyped	1	2.1
	Total	48	100

Chi-square value = 116.588, *P* < 0.0001.

### Congo-red binding assay

In the present study, 83.3% of the examined isolates (40/48) were positive for CR-binding assay. All the tested serovars were positive, including; O1 (9/48), O114 (7/48), O111 (5/48), O18 (4/48), O26 (4/48), O55 (4/48), O86a (4/48), and O158 (3/48), while the untyped strains were negative (8/48).

### Antimicrobial-resistance traits of the recovered isolates

**Figure 3:**
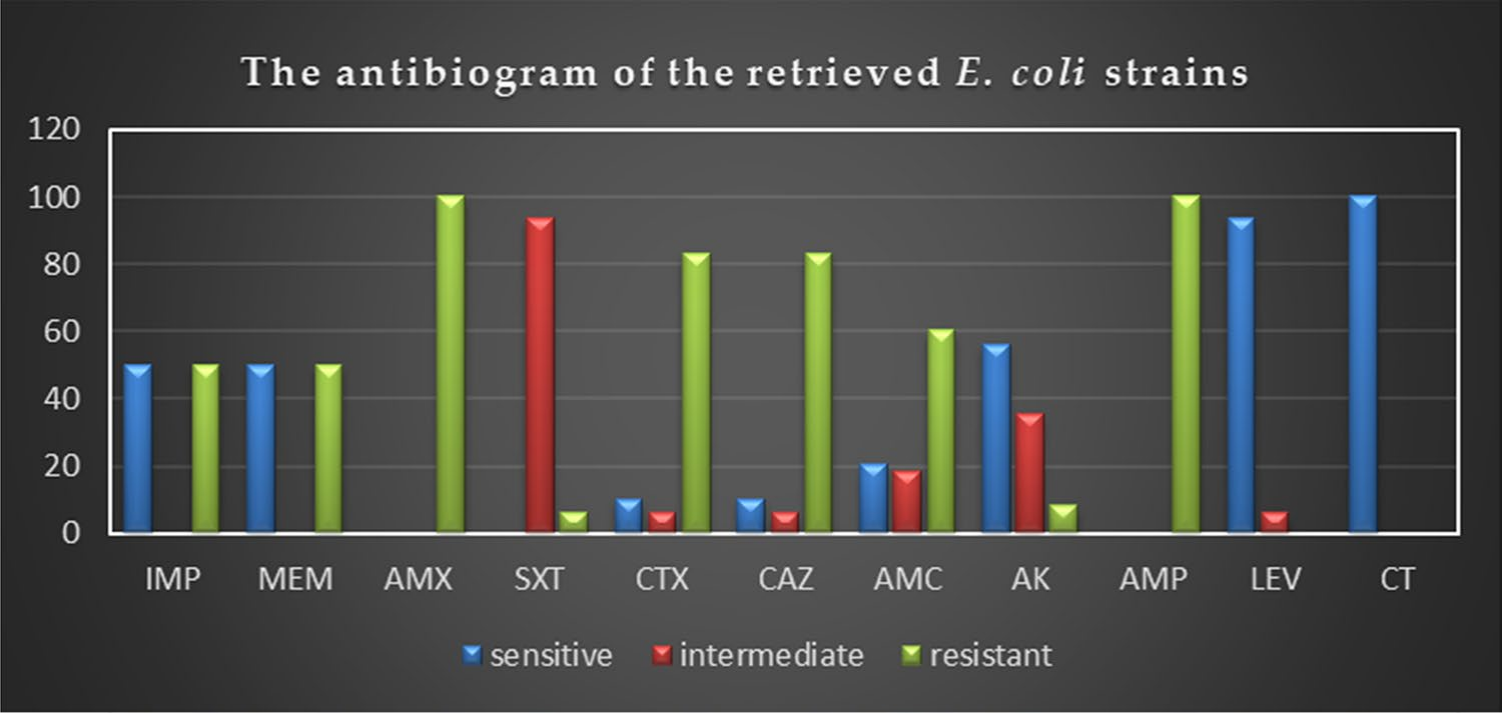
Antimicrobial resistance pattern of the retrieved *E. coli* strains (*n* = 48). The retrieved isolates displayed high resistance to ampicillin and amoxicillin (100%), cefotaxime and ceftazidime (83.3%), amoxicillin-clavulanic acid (60.4%), and imipenem and meropenem (50%). Besides, the examined strains were highly susceptible to colistin sulfate (100%), and levofloxacin (93.8%).

**Figure 4:**
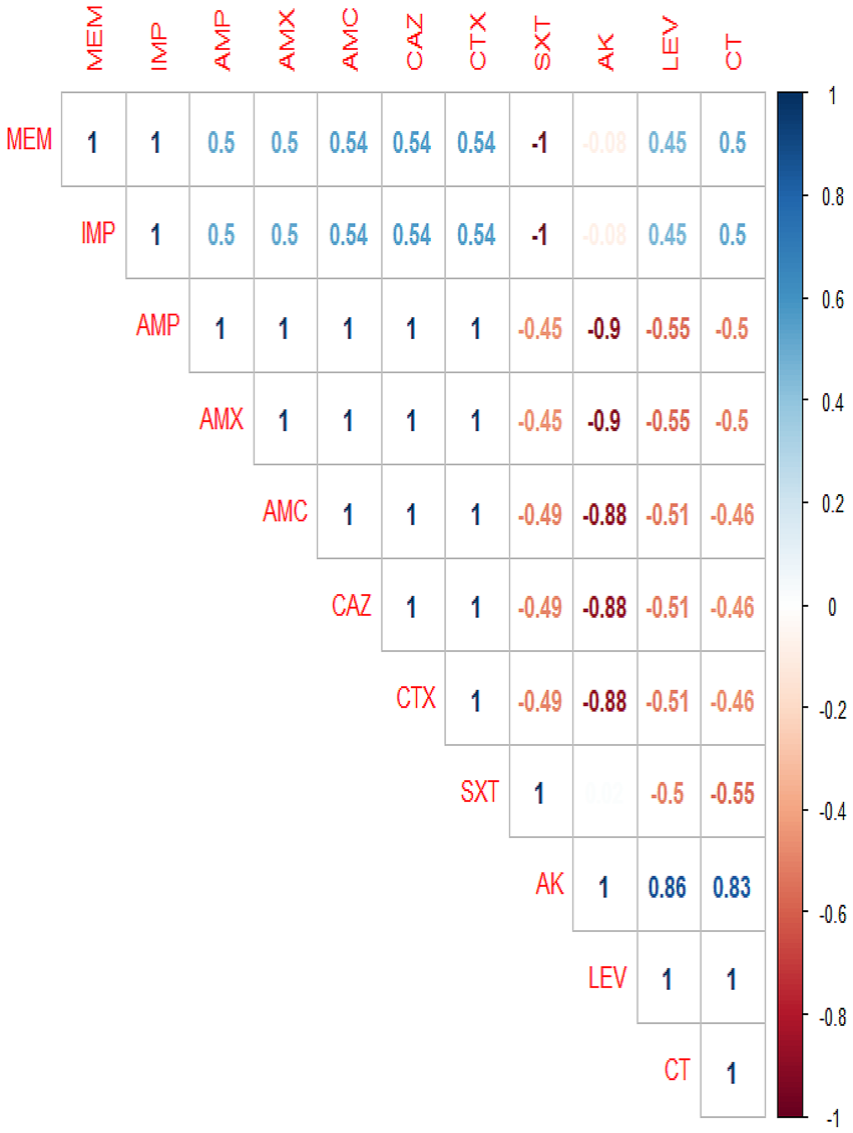
The correlation between the tested antimicrobial agents. The intensity of colors indicates the numerical value of the correlation coefficient (r), red, and blue color refers to the negative and positive correlations, respectively.

The in-vitro antimicrobial susceptibility testing revealed that the retrieved isolates displayed high resistance pattern to penicillins: ampicillin and amoxicillin (100%), and amoxicillin-clavulanic acid (60.4%), cephalosporins: cefotaxime and ceftazidime (83.3%), and carbapenems: imipenem and meropenem (50%), while showed intermediate resistance to trimethoprim-sulfamethoxazole (93.8%). Besides, the examined strains were highly susceptible to colistin sulfate (100%), followed by levofloxacin (93.8%) and amikacin (56.2%) as described in Table 4 and Fig. 3. Statistically, there is a significant difference in the resistance of the retrieved isolates to various tested antimicrobial agents (*P* < 0.05). The correlation analysis among the tested antimicrobial-agents was conducted. Our results revealed strong positive correlations (r = 0.5–0.86) between: AK and LEV (r = 0.83); AK and CT (r = 0.86); IMP, MEM, and CT (r = 0.5); IMP, MEM, and AMX (r = 0.5); IMP, MEM, and AMP (r = 0.5); IMP, MEM, and AMC (r = 0.54); IMP, MEM, and CAZ (r = 0.54); IMP, MEM, and CTX (r = 0.54). Furthermore, a moderate positive correlation was noticed between IMP and MEM (r = 0.45) (Fig. 4).

**Table 4:** Antimicrobial resistance pattern of the retrieved *E. coli* strains (*n* = 48).

Antibiotic classes	Specific tested antibiotic	Interpretation					
		Sensitive		Intermediate		Resistance	
		*N*	%	*N*	%	*N*	%
Penicillins	Amoxicillin	–	–	–	–	48	100
	Ampicillin	–	–	–	–	48	100
	Amoxicillin-Clavulanic acid	10	20.8	9	18.7	29	60.4
Cephalosporins	Cefotaxime	5	10.4	3	6.2	40	83.3
	Ceftazidime	5	10.4	3	6.2	40	83.3
Carbapenems	Imipenem	24	50	–	–	24	50
	Meropenem	24	50	–	–	24	50
Aminoglycosides	Amikacin	27	56.2	17	35.4	4	8.4
Fluoroquinolones	Levofloxacin	45	93.8	3	6.2	–	–
Polymyxins	Colistin sulfate	48	100	–	–	–	–
Sulfonamides	Trimethoprim-sulfamethoxazole	–	–	45	93.8	3	6.2
*P* value		*P* < 0.0001		*P* < 0.0001		*P* < 0.0001

### The frequency of the virulence-determinant and antibiotic-resistance genes among the recovered strains (n = 48)

**Figure 5:**
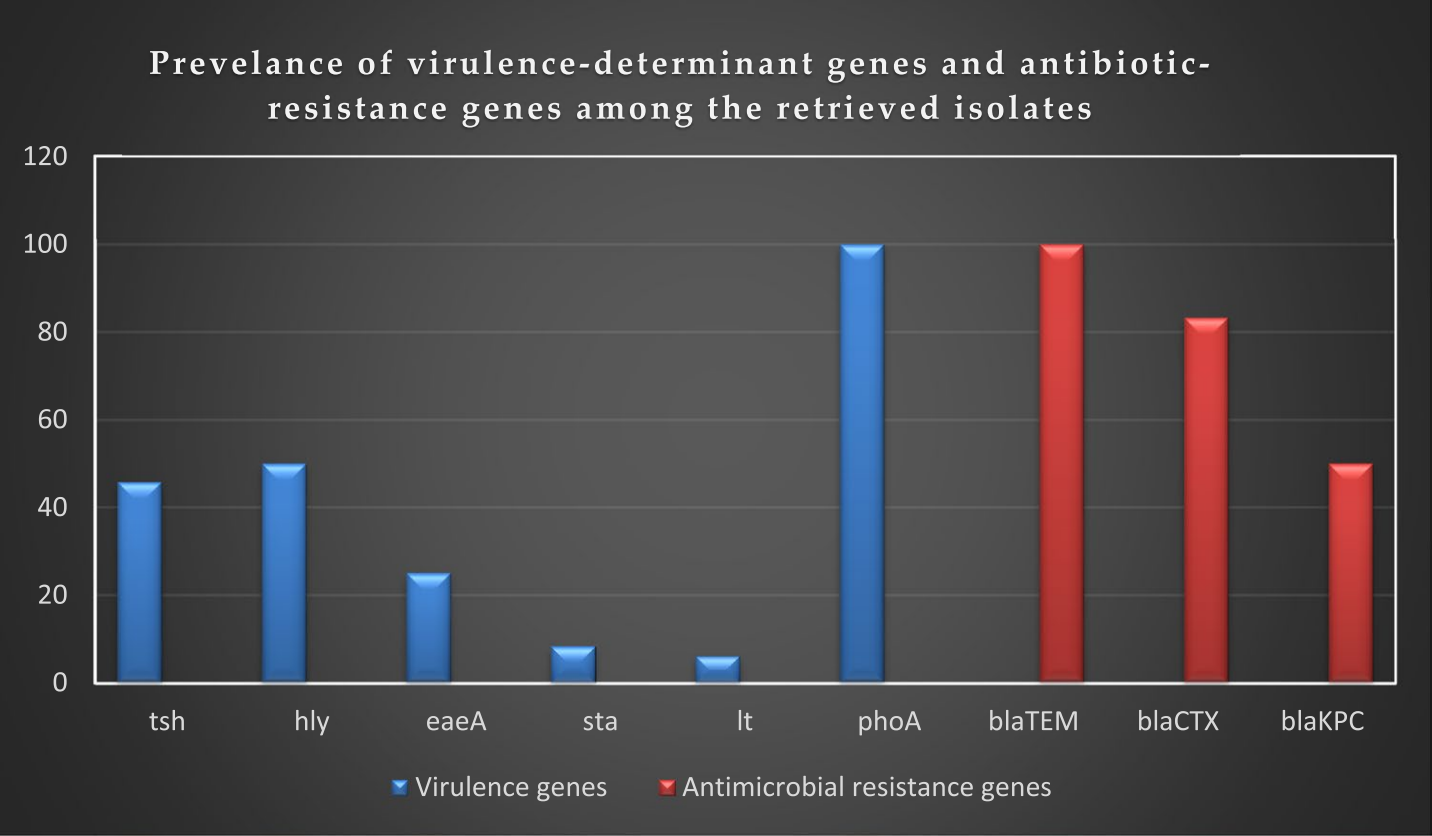
The distribution of virulence-determinant and antibiotic-resistance genes among the recovered strains. The tested strains harbored the virulence-determinant genes *pho*A, *hly*, *tsh*, *eae*A, *sta*, and *lt* with a prevalence of 100%, 50%, 45.8%, 25%, 8.4%, and 6.2%, respectively. Besides, they harbored the *bla*_TEM_, *bla*_CTX_, and *bla*_KPC_ resistance genes with a prevalence of 100%, 83.3%, and 50%, respectively.

Regarding the virulence-determinant genes, the PCR proved that the tested strains harbored the virulence genes *pho*A, *hly*, *tsh, eae*A, *sta*, and *lt* with a prevalence of 100% and 50%, 45.8%, 25%, 8.4%, and 6.2%, respectively. Concerning the antibiotic-resistance genes, the examined strains were positive for *bla*_TEM_, *bla*_CTX_, and *bla*_KPC_ resistance-genes with a prevalence of 100%, 83.3%, and 50%, respectively, as presented in Table 5. The frequency of the virulence-determinant and antibiotic-resistance genes in the retrieved serovars is illustrated in Tables 5 and 6, and Fig. 5. Statistically, there is a significant difference in the prevalence of the virulence-determinant genes and the antibiotic-resistant genes among the tested strains (*P* < 0.05).

**Table 5:** PCR-based screening of virulence and antibiotic resistance genes among the recovered strains (*n* = 48).

Target genes		*N*	%	*P* value
**Virulence-determinant genes**	*pho*A	48	100	*P* < 0.0001
	*hly*	24	50	
	*tsh*	22	45.8	
	*eae*A	12	25	
	*sta*	4	8.4	
	*lt*	3	6.2	
**Antibiotic-resistance genes**	*bla*_TEM_	48	100	*P* < 0.0001
	*bla*_CTX_	40	83.3	
	*bla*_KPC_	24	50

**Table 6:** Prevalence of virulence genes and antibiotic resistance genes among the retrieved serovars (*n* = 48).

Samples	Serovars	*N*	*tsh* gene	*pho*A gene	*hly* gene	*eae*A gene	*sta* gene	*lt* gene	*bla*_CTX_ gene	*bla*_KPC_ gene	*bla*_TEM_ gene
Nasal swabs	O86a	4	–	4	4	–	–	–	4	4	4
	Untyped	3	–	3	–	–	–	–	2	1	3
Fecal swabs	O114	7	7	7	1	3	4	–	5	5	7
	O26	4	–	4	4	1	–	3	4	4	4
	Untyped	2	–	2	–	2	–	–	2	–	2
Blood samples	O111	5	5	5	5	–	–	–	4	4	5
	O18	4	–	4	2	–	–	–	4	–	4
	Untyped	2	2	2	1	–	–	–	1	–	2
Milk samples	O1	9	2	9	3	5	–	–	7	2	9
	O55	4	3	4	2	–	–	–	3	4	4
	O158	3	2	3	2	–	–	–	3	–	3
	Untyped	1	1	1	–	1	–	–	1	–	1
Total		48	22	48	24	12	4	3	40	24	48

**Figure 6:**
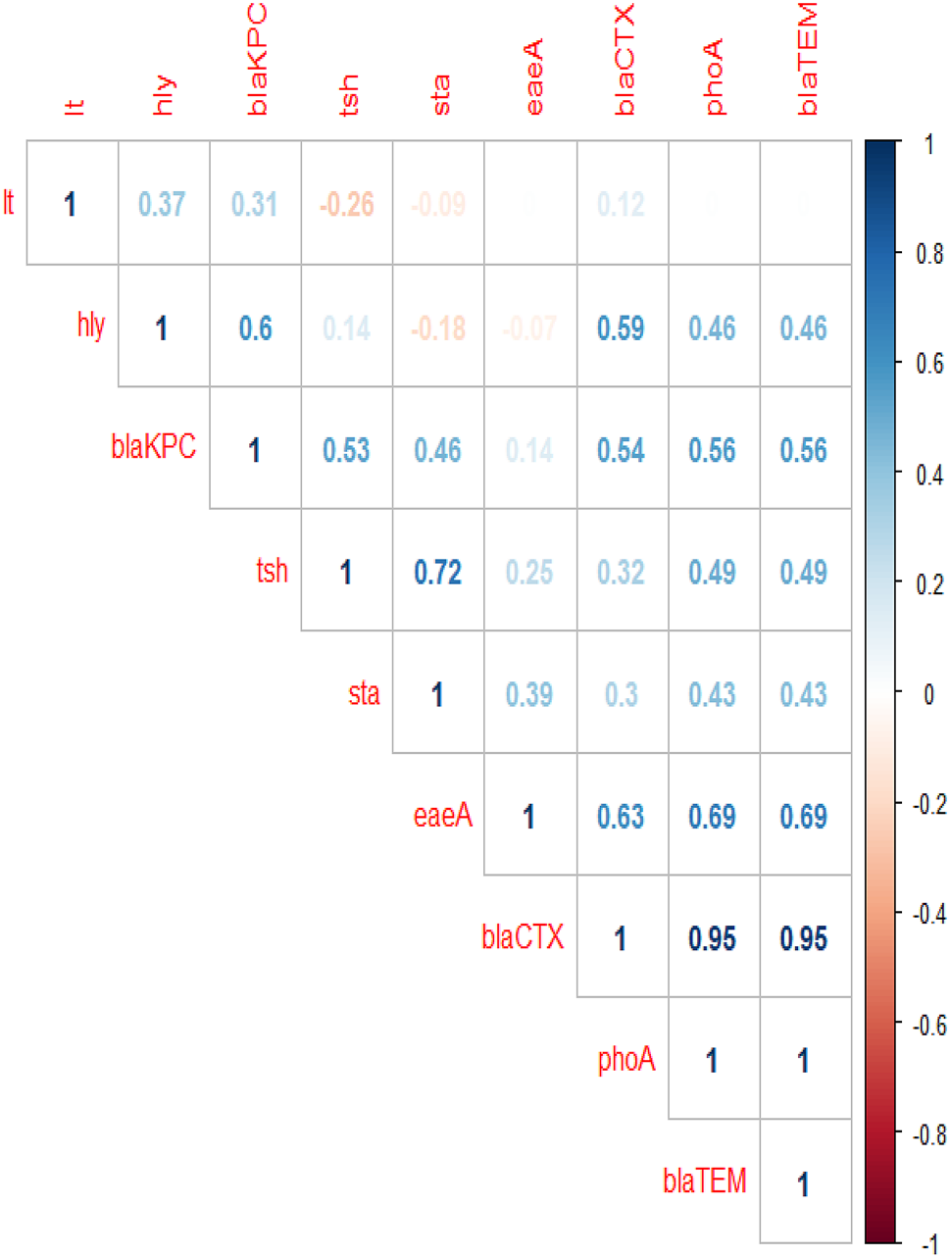
The correlation between virulence genes and the antibiotic-resistance genes. The intensity of colors indicates the numerical value of the correlation coefficient (r), red, and blue color refers to the negative and positive correlations, respectively.

**Figure 7:**
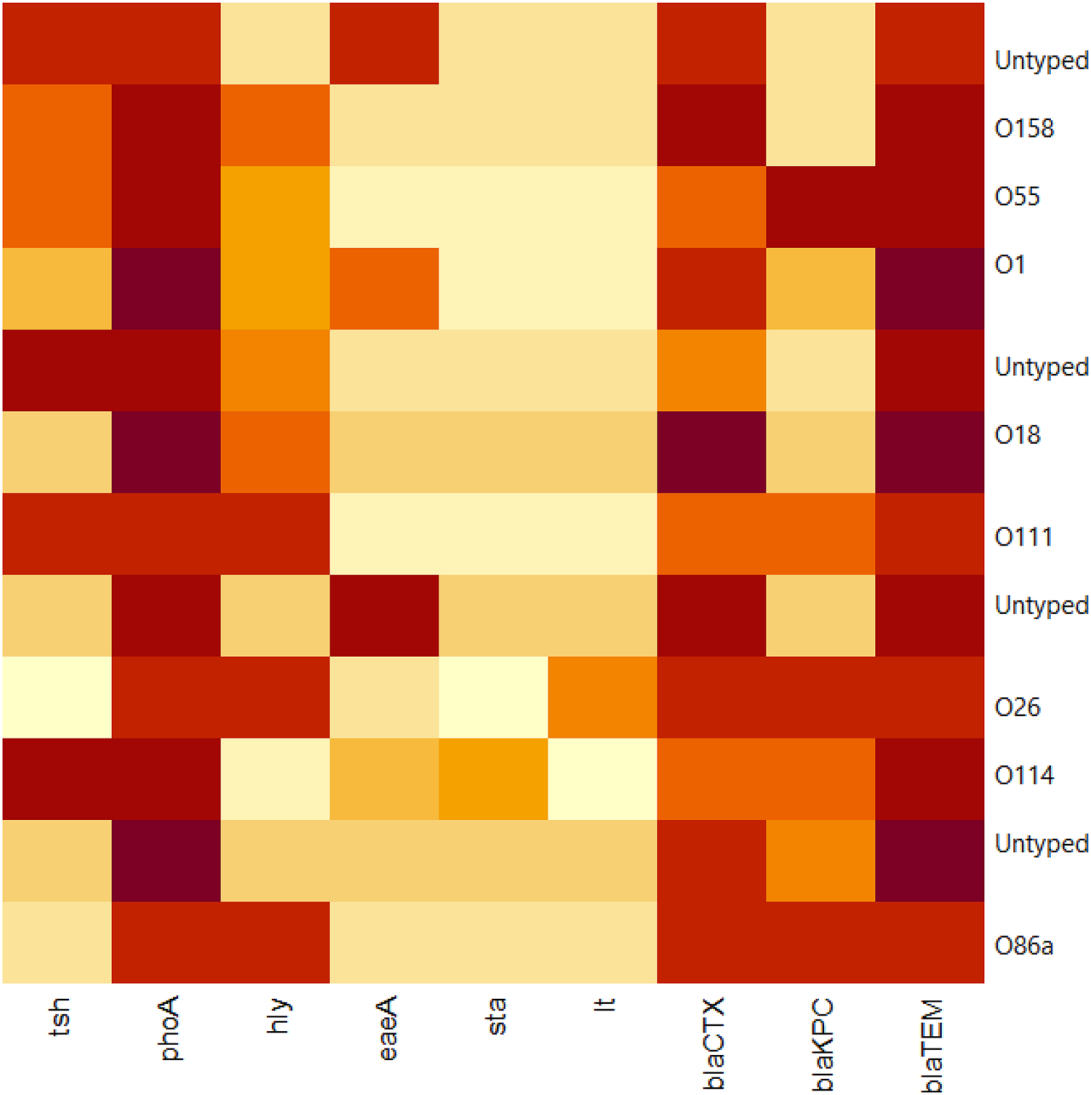
The heat-map illustrates the distribution of virulence genes and the antibiotic-resistance genes among the recovered *E. coli* serovars. The intensity of colors indicates the numerical value of the distribution.

The correlation analysis was determined between various virulence genes and antibiotic-resistance genes. The obtained results revealed strong positive correlations (r = 0.53–0.95) between: *bla*_CTX_, *bla*_TEM_, and *pho*A (r = 0.95); *tsh* and *sta* (r = 0.72); *eae*A, *bla*_TEM_, and *pho*A (r = 0.69); *eae*A and *bla*_CTX_ (r = 0.63), *hly* and *bla*_KPC_ (r = 0.6); *hly* and *bla*_CTX_ (r = 0.59); *bla*_KPC,_ *bla*_TEM_, and *pho*A (r = 0.56); *bla*_KPC_ and *bla*_CTX_ (r = 0.54); *bla*_KPC_ and *tsh* (r = 0.53). Moreover, moderate positive correlation (r = 0.3–0.49) was observed between: *tsh*, *bla*_TEM_, and *pho*A (r = 0.49); *hly*, *bla*_TEM_, and *pho*A (r = 0.46); *bla*_KPC_ and *sta* (r = 0.46); *sta*, *bla*_TEM_, and *pho*A (r = 0.43); *eae*A and *sta* (r = 0.39); *lt* and *hly* (r = 0.37); *bla*_CTX_ and *tsh* (r = 0.32); *lt* and *bla*_KPC_ (r = 0.31); *bla*_CTX_ and *sta* (r = 0.3). Besides, low positive correlation was noticed between *eae*A and *tsh* (r = 0.25) (Fig. 6). Furthermore, the heat-map illustrates the distribution of virulence genes and the antibiotic-resistance genes among the recovered *E. coli* serovars. The intensity of colors indicates the numerical value of the distribution (Fig. 7).

### The in-vitro multidrug-resistance patterns and the distribution of antibiotic-resistance genes

**Figure 8:**
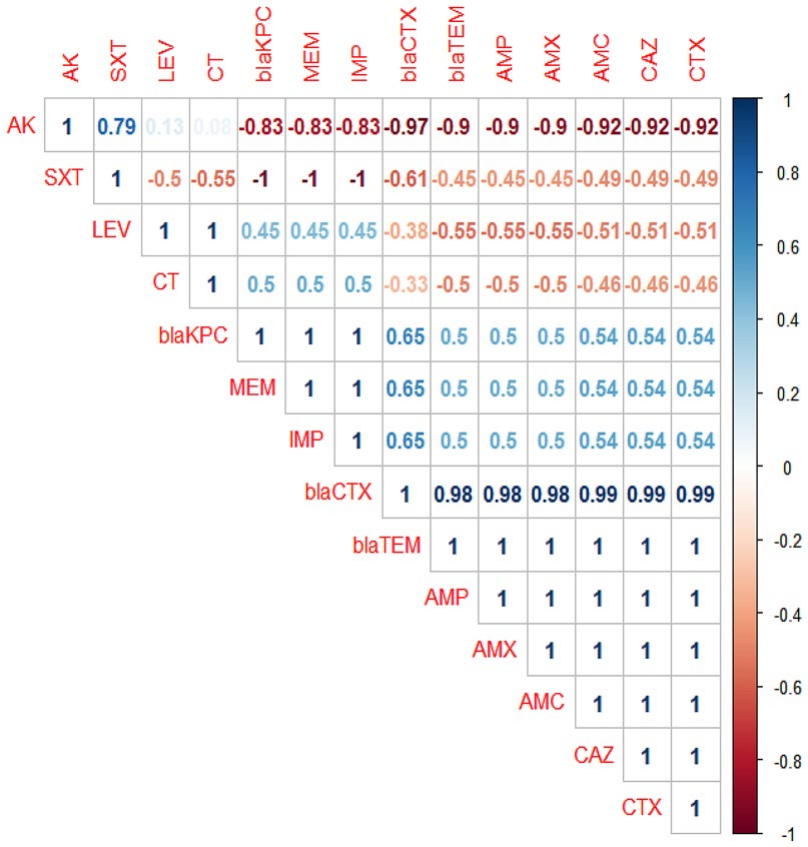
The correlation between various phenotypic multidrug-resistance patterns and the antibiotic-resistance genes. The intensity of colors indicates the numerical value of the correlation coefficient (r), red, and blue color refers to the negative and positive correlations, respectively.

Concerning the occurrence of multidrug-resistance phenomena, in the present study, 50% of the recovered strains are multidrug-resistant (MDR) (MDR: non-susceptible to ≥ one agent in ≥ three antimicrobial classes); to penicillins: ampicillin, amoxicillin, and amoxicillin–clavulanic acid; cephalosporins: ceftazidime and cefotaxime; carbapenems: meropenem and imipenem, and are harboring the *bla*_TEM_*, bla*_CTX_, and *bla*_KPC_ genes*.* Moreover, 25% of the examined strains are resistant to penicillins: ampicillin, amoxicillin, and cephalosporins: ceftazidime, and cefotaxime, and are harboring the *bla*_TEM_ and *bla*_CTX_ genes. Furthermore, 8.3% of the recovered strains were multidrug-resistant (MDR) to penicillins: ampicillin, and amoxicillin; cephalosprins: ceftazidime, cefotaxime, and aminoglycosides: amikacin, and possessed the *bla*_TEM_ and *bla*_CTX_ resistance genes (Table 7). The correlation analysis performed between various phenotypic multidrug-resistance patterns and the antibiotic-resistance genes. The obtained results revealed strong positive correlations between: *bla*_CTX_ gene, CAZ, and CTX (r = 0.99); *bla*_TEM_ gene, AMX, AMP, and AMC (r = 1); *bla*_KPC_ gene, MEM, and IMP (r = 1) (Fig. 8).

**Table 7:** The frequency of the phenotypic multidrug-resistance and the antibiotic-resistance genes among the retrieved strains (*n* = 48).

No. of strains	%	Type of resistance	Phenotypic multidrug resistance	The antibiotic -resistance genes
24	50	MDR	Penicillins: ampicillin, amoxicillin, amoxicillin–clavulanic acid	*bla*_TEM_*, bla*_CTX_, and *bla*_KPC_
			Cephalosporins: cetazidime, cefotaxime	
			Carbapenems: meropenem and imipenem	
12	25	Resistant	Penicillins: ampicillin and amoxicillin	*bla*_TEM_ and *bla*_CTX_
			Cephalosporins: cetazidime and cefotaxime	
4	8.3	MDR	Penicillins: ampicillin and amoxicillin	*bla*_TEM_ and *bla*_CTX_
			Cephalosporins: cetazidime and cefotaxime	
			Aminoglycosides: amikacin	
3	6.3	Resistant	Penicillins: ampicillin, amoxicillin, amoxicillin–clavulanic acid	*bla*_TEM_
			Sulfonamides: trimethoprim–sulfamethoxazole	
3	6.3	Resistant	Penicillins: ampicillin, and amoxicillin	*bla*_TEM_
2	4.1	Resistant	Penicillins: ampicillin, amoxicillin, and amoxicillin–clavulanic acid	*bla*_TEM_

Characteristics of multidrug resistance (MDR), extensively drug-resistance (XDR), and pandrug-resistance (PDR) in *E. coli:* *PDR* non-susceptible to all antimicrobial agents listed, *XDR* non-susceptible to ≥ one agent in all but ≤ two antimicrobial classes, *MDR* non-susceptible to ≥ one agent in ≥ three antimicrobial classes.

## Discussion

Globally, cattle are representing the main supply of high-quality meat and milk. However, few reports explained the role of pathogenic *E. coli* as a secondary bacterial pathogen following the FMD-outbreaks. The current study was conducted to inspect the prevalence, antibiogram, PCR detection of virulence-determinant genes (*tsh*, *pho*A, *hly*, *eae*A, *sta*, and *lt*) and the antibiotic-resistance genes (*bla*_TEM_, *bla*_KPC_, and *bla*_CTX_) of *E. coli* isolated from secondary bacterial infections following FMD-outbreak in cattle.

The bacteriological assay proved that *E. coli* was detected in 30% of the examined samples. Besides, other bacterial pathogens were isolated from 112 (70%) examined diseased animals. There is no significant difference in the prevalence of *E. coli* between the surveyed farms (*P* > 0.05), as the inspected farms are very close to each other and sharing the same management practices, nutrition, and water supply. *E. coli* is a common opportunistic microorganism that incriminated in several infections, especially diarrhea, mastitis, septicemia, and respiratory manifestations^11, 18, 23^. In Nigeria, *S. uberis* and *S. bovis* clinical mastitis are also reported by Amosun^31^. In China, the emergence of *P. mirabilis* as a causative agent of diarrhea was reported by Gong^32^. Moreover, in Nepal, *E. faecalis* diarrhea was recorded in immune-compromised persons by Sah^33^. El-Seedy^34^ reported that *P. multocida* and *M. hemolytica* are major pathogens of calf pneumonia in Egypt, while Algammal^11^ categorized *P. aeruginosa* as a common pathogen of pneumonia in calves. In Egypt, although the available FMD-vaccine is efficient to minimize the mortality rate, the vaccination-failure may happen that results in the occurrence of FMD-outbreak and the emergence of secondary bacterial infections due to the immunosuppression^35^. A previous study in Cambodia reported the occurrence of FMD-vaccination failure in more than 50% of the vaccinated animals. The vaccination failure is mainly attributed to improper technique, insufficient dose, immunological factors, and vaccine cold-chain miscarriage^36^. Several causes are implicated in the existence of *E. coli* secondary infection, including; bad sanitation, intensive-breeding management, bad environmental conditions, stress, and weak animal immunity^18^.

Concerning the *E. coli* serovars, the most prevalent *E. coli* serovar accompanied the respiratory infection was 086a (n = 4), diarrhea: O114 (n = 7), fever: O111 (n = 5), mastitis: O1 (n = 9). The investigation of *E. coli* O-serogroups has a major public health concern. The recovered serovars are analogous to those reported by previous studies, which concerned the *E. coli* infections^37–39^. In the present study, the CR-binding assay proved that 83.3% of the examined isolates (40/48) were CR-binding positive. All the tested serovars were positive. Moreover, the untypable strains were negative (8/48). The current results agreed with Algammal^18^, who reported that 89.8% of the tested strains are invasive by congo-red binding assay, which confirms the pathogenicity of these isolates.

Regarding the in-vitro antimicrobial susceptibility testing, the retrieved strains exhibited a remarkable resistance to penicillins, cephalosporins, and carbapenems which gave a public health alarm. The current findings nearly agreed with those reported by Shahrani^40^, Gupta^41^, and Touwendsida^42^. The uncontrolled widespread use of antibiotics in veterinary and health sectors as well as the bacterial antibiotic-resistant genes are incriminated in the development of such multidrug-resistant strains^43, 44^. Regrettably, *E. coli* is capable to resist various antibiotic-classes due to possessing resistant genes and/ or R-plasmids^45^.

In the current study, the PCR proved that the recovered *E. coli* strains were found to posse 2–5 virulence genes. The most prevalent virulence genes accompanied the respiratory infections are *pho*A and *hly* genes, in diarrhea: *pho*A*, sta, lt, eae*A*,* and *hly* genes, in fever and mastitis: *pho*A*, tsh,* and *hly* genes. These findings agreed with those obtained by previously reported by Algammal^18^, Andrade^46^ , and Whitelegge^47^. The pathogenesis of virulent *E. coli* is controlled by multiple virulence determinants that vary among different pathotypes. The most common virulence determinants that accompanied the *E. coli*-pathotypes are enterotoxins, hemolysins, siderophores, intimin, fimbria-mannose binding type1-H adhesion, alkaline phosphatase, and temperature-sensitive haemagglutinin (Tsh-protein). Furthermore, the production of these virulence-determinants is regulated by the expression of specific virulence genes^48, 49^.

In the present study, 50% of the recovered strains are MDR to penicillins, cephalosporins, and carbapenems, and are harboring the *bla*_TEM_*, bla*_*CTX*_, and *bla*_KPC_ genes*.* Furthermore, 25% of the examined strains are resistant to penicillins and cephalosporins, and are harboring the *bla*_TEM_ and *bla*_CTX_ genes. The Extended Spectrum β-lactamases (ESBLs) produced by *E. coli* incriminated in the β-lactam-antibiotic resistance. The heavy use of penicillin, cephalosporins, and carbapenems-antibiotics in medications is resulting in the evolution of multidrug-resistant strains. The resistance to the β-lactam-antibiotics is mainly mediated by the ESBL-genes; *bla*_TEM_, *bla*_CTX_, and *bla*_KPC_ which are encoded for penicillin, cephalosporins, and carbapenem-resistance, respectively^50–53^. Different mechanisms explain the emergence of MDR-*E. coli* strains include: 1-Shared resistance mechanisms; occur especially for the antimicrobial agents in the same category due to penicillin-binding protein mutations as well as the β-lactamases. Furthermore, it could happen for different antibiotics in various classes due to the efflux pumps acting on numerous drugs in different species. 2-Linkage among the antibiotic resistance genes, this mechanism plays a significant role in association links between various resistances and to differentiate between resistance mechanisms (either the resistance arise due to alterations in the target protein of the antibiotic or due to a resistance gene encoded for an enzyme that destroys the antibiotic). 3-Correlated drug exposure of the host, it mainly occurs due to routine use of combination therapy and the repeated treatment failure^54–59^.

Limitations and future recommendations: Future work is recommended to perform phylogenetic analysis either by MLST or PFGE to understand the clonal relatedness of the obtained strains.

In conclusion, to the best of our knowledge, this is the first report concerning the *E. coli* secondary bacterial infections following the FMD-outbreak. The immunosuppression due to the FMD increases the animal susceptibility to *E. coli* secondary infections. The most prevalent *E. coli* serovar associated the respiratory infections was O86a, in diarrhea: O114, in fever: O111, and in mastitis: O1. Furthermore, the most predominant virulence-determinant genes accompanied the *E. coli* respiratory infections were *pho*A and *hly* genes*,* diarrhea: *pho*A*, sta*, *lt*, *eae*A, and *hly* genes*,* fever, and mastitis: *pho*A, *tsh*, and *hly* genes. A high percentage of the isolated *E. coli* strains were multidrug resistant (MDR) to penicillins: ampicillin, amoxicillin, and amoxicillin-clavulanic acid; cephalosporins: ceftazidime and cefotaxime; carbapenems: meropenem and imipenem, and are harboring the *bla*_TEM_, *bla*_*CTX*_, and *bla*_KPC_ genes. In-vitro, colistin sulfate and levofloxacin have promising activity against MDR-*E. coli*. The emergence of highly pathogenic MDR-*E. coli* strains constitutes a significant threat to the cattle health resulting in multiple severe infections and huge economic losses in the livestock production. Furthermore, the evolution of penicillins, cephalosporins, and carbapenems-resistant strains is reflecting a public health alarm and specifies the convoluted treatment of the infections caused by these strains. Moreover, it recommends the proper use of antimicrobial agents in the veterinary and health sectors as well as the routine application of the antimicrobial susceptibility testing.
